# Genetic structuring among colonies of a pantropical seabird: Implication for subspecies validation and conservation

**DOI:** 10.1002/ece3.6635

**Published:** 2020-09-29

**Authors:** Laurence Humeau, Matthieu Le Corre, Silas James Reynolds, Colin Wearn, Janos C. Hennicke, James C. Russell, Yann Gomard, Hélène Magalon, Patrick Pinet, Pauline Gélin, François‐Xavier Couzi, Etienne Bemanaja, Vikash Tatayah, Bacar Ousseni, Gérard Rocamora, Patrick Talbot, Nirmal Shah, Leandro Bugoni, Denis Da Silva, Audrey Jaeger

**Affiliations:** ^1^ UMR PVBMT Université de La Réunion CIRAD Saint‐Denis Cedex 9 La Réunion France; ^2^ UMR ENTROPIE Université de La Réunion IRD CNRS Saint‐Denis Cedex 9 La Réunion France; ^3^ Centre for Ornithology School of Biosciences College of Life & Environmental Sciences University of Birmingham Birmingham UK; ^4^ Army Ornithological Society (AOS) c/o Prince Consort Library Aldershot Hampshire UK; ^5^ Royal Air Force Ornithological Society (RAFOS) Royal Air Force Headquarters Buckinghamshire UK; ^6^ Department of Ecology and Conservation Biocentre Grindel University of Hamburg Hamburg Germany; ^7^ Centre d'Etudes Biologiques de Chizé CEBC‐CNRS Villiers‐en‐Bois France; ^8^ School of Biological Sciences and Department of Statistics University of Auckland Auckland New Zealand; ^9^ UMR PIMIT CNRS INSERM IRD Université de La Réunion Plateforme Technologique CYROI Sainte‐Clotilde La Réunion France; ^10^ Parc National de La Réunion La Plaine des Palmistes La Réunion France; ^11^ Société d'Etudes Ornithologiques de La Réunion (SEOR) Saint André La Réunion France; ^12^ Centre National de Recherches Océanographiques (CNRO) Nosy Be Madagascar; ^13^ Mauritian Wildlife Foundation Vacoas Mauritius; ^14^ GEPOMAY M'tsangamouji Mayotte France; ^15^ Island Conservation Society Mahé Seychelles; ^16^ Island Biodiversity and Conservation centre University of Seychelles Mahé Seychelles; ^17^ Bermuda Zoological Society Flatts Bermuda; ^18^ Nature Seychelles The Center for Environment and Education Mahé Seychelles; ^19^ Instituto de Ciências Biológicas Universidade Federal do Rio Grande (FURG) Rio Grande Brazil

**Keywords:** conservation status, genetic structure, *Phaethon lepturus*, subspecies status

## Abstract

Investigations of the genetic structure of populations over the entire range of a species yield valuable information about connectivity among populations. Seabirds are an intriguing taxon in this regard because they move extensively when not breeding, facilitating intermixing of populations, but breed consistently on the same isolated islands, restricting gene flow among populations. The degree of genetic structuring of populations varies extensively among seabird species but they have been understudied in their tropical ranges. Here, we address this across a broad spatial scale by using microsatellite and mitochondrial data to explore the population connectivity of 13 breeding populations representing the six subspecies of the white‐tailed tropicbird (*Phaethon lepturus*) in the Atlantic, Indian, and Pacific Oceans. Our primary aim was to identify appropriate conservation units for this little known species. Three morphometric characters were also examined in the subspecies. We found a clear pattern of population structuring with four genetic groups. The most ancient and the most isolated group was in the northwestern Atlantic Ocean. The South Atlantic populations and South Mozambique Channel population on Europa were genetically isolated and may have had a common ancestor. Birds from the Indo‐Pacific region showed unclear and weak genetic differentiation. This structuring was most well defined from nuclear and mtDNA markers but was less well resolved by morphological data. The validity of classifying white‐tailed tropicbirds into six distinct subspecies is discussed in light of our new findings. From a conservation standpoint our results highlight that the three most threatened conservation units for this species are the two subspecies of the tropical North and South Atlantic Oceans and that of Europa Island in the Indian Ocean.

## Graphical abstract

Morphology and population genetic structure of the white‐tailed tropicbird (*Phaethon lepturus*) over its entire range were analyzed in order to investigate population connectivity and relatedness. Our results are an example of how thorough genetic analyses can reveal critical information about subspecies delimitation and conservation priorities.

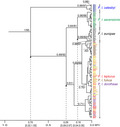

## 1 INTRODUCTION

Widely distributed species consist of distinct populations that are variously connected to each other though current and historic overlaps in ranges, resulting in different patterns of genetic structuring at global and local scales (Avise & Ball, 1990; Frankham, Ballou, & Briscoe, 2004). In mobile animals, life‐history traits such as dispersal and philopatry, as well as geographic isolation, shape population genetic connectivity (Dobzhansky & Dobzhansky, 1970; Greenwood, 1980; Matthiopoulos, Harwood, & Thomas, 2005; Taylor & Friesen, 2012; Wright, 1943). Many species with wide distribution are subdivided into subspecies (Ball & Avise, 1992). The distinction between a subspecies and a species is at time far from clear but a lineage can be considered as a new species when it acquires sufficiently different properties from others. Such differences can include being phenetically distinguishable, diagnosable, reciprocally monophyletic, reproductively incompatible, and ecologically distinct (de Queiroz, 2007). There is a critical (and ongoing) need for delineation of species and subspecies because it is fundamental in elucidating key processes in ecology (e.g., population connectivity) and conservation biology (e.g., definition of conservation priority units; Dayrat, 2005; Gutiérrez & Helgen, 2013). Traditional morphology‐based taxonomy defining “morphotypes” (i.e., taxa described solely from morphological traits) faces challenges when applied to taxa that are either cryptic (i.e., a group of individuals that is morphologically indistinguishable but incapable of interbreeding) or display considerable phenotypic plasticity. Therefore, Zink (2004) and Sackett et al. (2014) argued that only taxa defined by the congruence of multiple morphological or molecular characters should be recognized as distinct subspecies.

Investigating genetic population structure over the entire range of a species, with highly variable DNA markers such as microsatellites and mitochondrial DNA (mtDNA), provides valuable insights into speciation, population connectivity, biogeography, genetic drift, and within‐population genetic diversity (Burg & Croxall, 2001; Godinho, Crespo, & Ferrand, 2008; Thanou et al., 2017). These genetic analyses also provide valuable information to identify appropriate conservation units allowing implementation of adaptive conservation plans for threatened species, subspecies, or populations (Dayrat, 2005; Fraser & Bernatchez, 2001; Gutiérrez & Helgen, 2013).

Seabirds are an intriguing taxon when their populations are considered in terms of gene flow and genetic diversity and structuring. Despite having wide dispersal potential over entire ocean basins (Booth Jones et al., 2017; Egevang et al., 2010; González‐Solís, Croxall, Oro, & Ruiz, 2007), they often show high levels of population isolation and even endemism. This is the so‐called “seabird paradox” (Friesen, 2015; Lombal, O'dwyer, Friesen, Woehler, & Burridge, 2020; Milot, Weimerskirch, & Bernatchez, 2008; Welch et al., 2012).

Of the three species of tropicbird (Phaethontidae), the white‐tailed tropicbird *Phaethon lepturus* is the most common (Lee & Walsh‐McGehee, 1998). This pantropical seabird is widely distributed in the Atlantic, Pacific, and Indian Oceans between 30°N and 30°S (del Hoyo, Elliott, Sargatal, & Christie, 1992). Although its global conservation status is of “Least Concern,” the white‐tailed tropicbird is decreasing globally predominantly because of predation by invasive species (BirdLife International, 2018). Declining populations are reported in the Atlantic islands where tropicbirds have been depredated by introduced brown rats *Rattus norvegicus*, black rats *R. rattus*, feral domestic cats *Felis sylvestris catus*, dogs *Canis lupus familiaris*, American Crows *Corvus brachyrhynchos* (on Bermuda), and tegu lizards *Salvator merianae* (in the Fernando de Noronha archipelago) and where seabirds generally have long endured persecution from humans (Efe, Serafini, & Nunes, 2018; Lee & Walsh‐McGehee, 2000; Nunes, Efe, Freitas, & Bugoni, 2017). In Bermuda, one of the main threats was habitat destruction through storm damage; in 2003, a category 3 hurricane damaged 50% of natural nest sites of white‐tailed tropicbirds in one of national parks (PT unpublished data). In the Indian Ocean, the breeding tropicbird population of Aride Island, Seychelles, suffered a marked decline of 60% between 1989 and 1998 (Bowler, Betts, Bullock, & Ramos, 2002) which continues to the present day as a 5.4% annual rate of decline (Catry et al., 2009). Threats to tropicbirds on Aride have not been clearly identified to date but 23.2% of recorded adult mortalities are due to entanglement in *Pisonia* sticky seeds with birds losing the ability to fly off. This may be demographically significant (Catry et al., 2009).

**FIGURE 1:**
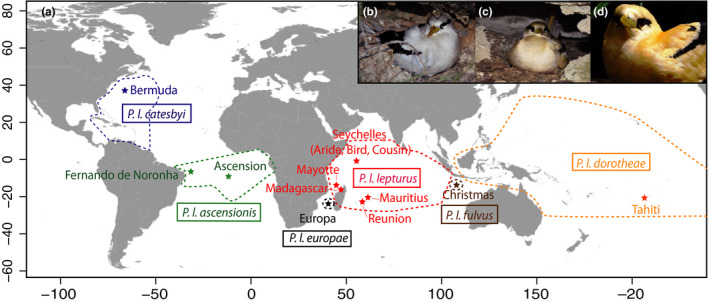
(a) Locations (stars) and associated subspecies of white‐tailed tropicbird populations sampled in the Atlantic, Indian and Pacific Oceans (see Tables 1 and 2 for further details). Range (dotted lines) of each subspecies is indicated (BirdLife International, 2020). Variation in plumage coloration variation among three subspecies of *Phaethon lepturus*: (b) white morph of *P. l. lepturus* from Mayotte Island (Photo credit: MLC), (c) yellow morph of *P. l. europae* from Europa Island (Photo credit: A. Laubin), and (d) apricot morph of *P. l. fulvus* from Christmas Island (Photo credit: PP)

Based on morphological differences, six subspecies of *P. lepturus* are currently recognized, three occurring in the Indian Ocean, two in the Atlantic Ocean, and one in the Pacific Ocean (del Hoyo et al., 1992; Le Corre & Jouventin, 1999). Three large subspecies breed in the western (*P. l. lepturus*) and eastern (*P. l. fulvus*) Indian Ocean and in the northwestern Atlantic Ocean (*P. l. catesbyi*). The three small subspecies breed in the Pacific (*P. l. dorotheae*), Indian (South Mozambique Channel; *P. l. europae*), and south Atlantic Oceans (*P. l. ascensionis*). The species has golden or apricot plumage morphs in some populations in which birds have white parts of the body plumage that are tinged with these shades. Populations of *P. l. fulvus* and *P. l. europae* comprise large fractions of apricot and golden birds, respectively, whereas other subspecies contain mainly white individuals (Le Corre & Jouventin, 1999; see Table 1 and Figure 1).

**TABLE 1:** Characteristics of the six subspecies of *Phaethon lepturus*

Subspecies	Distribution range	Breeding locations	Color morph	Body size group^a^
*catesbyi*	North Atlantic Ocean	Bermuda, Caraibes	White	Large
*ascensionis*	South Atlantic Ocean	Ascension, Sao Tomé, Fernando de Noronha	White	Small
*europae*	South Mozambique Channel	Single: Europa Island	Golden	Small
*lepturus*	West Indian Ocean	Three archipelagos (Seychelles, Comores, Mascarene), Madagascar	White	Large
*fulvus*	East Indian Ocean	Single: Christmas Island	Apricot	Large
*dorotheae*	Pacific Ocean	Numerous	White	Small

^a^ After Le Corre and Jouventin (1999).

Such body size and plumage differences among subspecies and locations suggest some level of genetic isolation among populations (Le Corre & Jouventin, 1999). In addition, even neighboring populations could have sharp genetic structuring (Nunes et al., 2017; Wiley et al., 2012). Therefore, the aim of the present study was to explore genetic diversity and population genetic structuring across the entire range of the white‐tailed tropicbird and to infer genetic relationships among populations. Both microsatellite and mtDNA markers were used to investigate genetic structure of 13 breeding populations of the Atlantic, Indian, and Pacific Oceans, including birds from all of the six recognized subspecies. We also performed morphometric analyses to examine patterns of phenotypic differentiation among 11 populations of birds for which data were available. Our findings allow us to discuss subspecies validity and appropriate units for effective conservation strategies.

## 2 MATERIALS AND METHODS

### 2.1 Study sites and sampling

Thirteen populations were sampled between 2008 and 2013 in Atlantic, Indian, and Pacific Oceans (see Table 2 and Figure 1 for further details). In each population (except that at Réunion Island), all birds were sampled from the same local area and thus can be considered to belong to the same breeding colony. On Réunion Island, we sampled birds from the local Wildlife Rescue Center (SEOR) that originated from various locations across the island. Details of field researchers and local licenses (when required) are provided in supporting information (Appendix S1, Table S1). Blood was collected from 382 birds by venepuncture of the brachial or metatarsal veins and samples were stored in 70% ethanol or by sterile syringe and needle and stored dry on filter paper.

**TABLE 2:** Characteristics of the studied populations of *Phaethon lepturus*

Region	Population	Code	Latitude	Longitude	Subspecies	*N*	Nmsat	Nmt DNA
North Atlantic	Bermuda	BER	32°18N	64°47W	*catesbyi*	11	21	8
South Atlantic	Ascension	ASC	7°57S	14°21W	*ascensionis*	13	20	14
	Brazil (Fernando de Noronha)	BRA	3°50S	32°24W	*ascensionis*	60	4	4
South Mozambique Channel	Europa	EUR	22°21S	40°22E	*europae*	50/140	50	14
West Indian	Mayotte	MAY	12°49S	45°10E	*lepturus*	40	47	13
	Madagascar (Nosy Hara)	MAD	13°19S	48°16E	*lepturus*	8	4	4
	Seychelles (Bird)	SEB	3°43S	55°12E	*lepturus*	32	34	7
	Seychelles (Cousin)	SEC	4°20S	55°40E	*lepturus*	144/230	69	12
	Seychelles (Aride)	SEA	4°13S	55°40E	*lepturus*	0	25	6
	Mauritius (Round Island)	MAU	20°20S	57°35E	*lepturus*	23/25	25	8
	Reunion	RUN	21°7S	55°31E	*lepturus*	41/45	55	13
East Indian	Christmas	CHR	10°29S	105°38E	*fulvus*	11/12	17	12
Pacific	Tahiti	TAH	17°39S	149°26W	*dorotheae*	0	11	8
	Overall					433/616	382	123

Note

*N*, Nmsat and NmtDNA: number of sampled individuals for morphometric and genetic (microsatellite and mtDNA) analyses, respectively.

### 2.2 Biometrics

Morphometric measurements were obtained from breeding adults in 11 populations (Table 2): (a) wing length (i.e., flattened wing from the carpal joint to the tip of the longest primary), (b) tarsus length (i.e., maximum tarsus length), and (c) bill length (i.e., exposed culmen) following the British Trust for Ornithology's (BTO’s) Ringers’ Manual (Redfern & Clark, 2001). Morphometric measurements of 616 individuals of white‐tailed tropicbirds are available in supporting information (Appendix S1, Table S2). Statistical differences in morphometric measurements of these birds between pairs of focal populations were examined using pairwise Wilcoxon rank sum tests corrected after Benjamini and Hochberg (1995), in R 3.4.2 (R Core Team, 2017). Global patterns in morphometric differences among birds and the populations to which they belong were investigated using a principal components analysis (PCA) performed on a correlation matrix of three morphometric measurements from 421 birds of 10 populations individuals for which we had measurements for all three characters (Appendix S1, Table S2) using the ade4 package in R (Dray & Dufour, 2007). Differences in the synthetic morphology of birds, as characterized from the three morphometric measurements, were investigated among the 10 populations of birds using a multivariate analysis of variance (MANOVA). The three principal component scores (i.e., PC1, PC2, and PC3) from the PCA were the variables in the MANOVA from which a Wilk's lambda (Huberty & Olejnik, 2006) was derived following 9,999 permutations. Benjamini and Hochberg (1995) corrected pairwise permutation MANOVAs were performed to compare morphometric measurements among pairwise populations, using the RVAideMemoire package in R (Hervé, 2019).

### 2.3 Microsatellite genotyping

Total DNA was extracted from blood or tissue samples using the QIAmp Blood and Tissue kit (Qiagen). Genotyping was conducted for 10 polymorphic microsatellite loci (described in Humeau et al., 2011) on DNA extracts from 382 individuals (Table 2). Amplified fragments were resolved by capillary electrophoresis on an automated sequencer ABI Prism 3100 Genetic Analyzer (Applied Biosystems). Allele sizes were determined using GeneMapper Software 4 with the LIZ500(‐250) standard (Applied Biosystems). Raw microsatellite genotypes of 382 individual white‐tailed tropicbirds are available in the supporting information (Appendix S1, Table S3).

### 2.4 Mitochondrial DNA sequencing

Mitochondrial DNA variation was analyzed in a subset of samples used for microsatellite genotyping (*n* = 123, Table 2) and was assayed following the amplification of part of the cytochrome oxidase subunit I (COI) region. Sequences from white‐tailed tropicbirds (Kennedy & Spencer, 2004; GenBank accessions AY369055, JN801349) were used to design two primers: CO1Fcons 5′‐GACCGAAACCTAAAYACCACA‐3′ and CO1Rcons 5′‐GGTTCGATTCCYTCCTTTCTT‐3′. Each PCR contained 3 µl of DNA solution (60 ng), 0.2 µM of each primer, 7.5 µl of Qiagen PCR MasterMix and was run on GeneAmp PCR System 9700 thermalcycler (Applied Biosystems). The PCR consisted of 35 cycles at 94°C for 30 s, 58°C for 90 s, and 72°C for 90 s, preceded by an initial denaturation step of 5 min at 94°C and followed by a 15 min final extension step at 72°C. DNA templates were purified and sequenced in both directions by Genoscreen (Lille, France), obtaining 872 bp sequences (GenBank accession numbers: KY784127–KY784144, and see Appendix S1, Table S4 for details). In addition to the *P. lepturus* samples, DNA from six red‐tailed tropicbirds of *P. rubricauda* was also included in the phylogenetic analysis as an outgroup (GenBank accession numbers: MT073267 and MT073268; Appendix S1, Table S4). This species is the phylogenetically closest interspecific relative to *P. lepturus* (Kennedy & Spencer, 2004).

### 2.5 Genetic diversity

Evidence of null alleles, large‐allele dropout, and stutter peaks in all microsatellites was examined using MicroChecker 2.2.3 (Van Oosterhout, Hutchinson, Wills, & Shipley, 2004). Each locus‐pair combination was tested for linkage disequilibrium with GenePop 4.0.10 (Rousset, 2008). The *p*‐values were corrected using the Benjamini and Yekutieli (2001) method for multiple comparisons (Narum, 2006). The mean observed number of alleles per locus (AL) and the number of private alleles per population (AP) were computed using GenAlEx 6.5 (Peakall & Smouse, 2012). Allelic richness (AR—El Mousadik & Petit, 1996), adjusted for discrepancies in sample size by incorporating a rarefaction method as implemented using FSTAT 2.9.3 (Goudet, 2001), was used to make comparisons of the mean number of alleles among populations (except for the populations from Fernando de Noronha and Madagascar that contained only four individuals each). Observed heterozygosity (*H*_O_), unbiased expected heterozygosity estimated according to Nei (1978) (*H*_E_), and Wright's *F*‐statistics *F*_IS_ according to the method of Weir and Cockerham (1984) were calculated for each population using GENETIX 4.05.2 (Belkhir, Borsa, Chikhi, Raufaste, & Bonhomme, 1996–2004). Deviations from Hardy–Weinberg equilibrium (HWE) were tested for each population using GENETIX 4.05.2.

To test whether patterns of genetic variation in the mtDNA sequences deviated from neutral expectation, Ewens–Watterson test (Ewens, 1972; Watterson, 1978) was performed in ARLEQUIN 3.5.1.3 (Excoffier & Lischer, 2010). Haplotypic diversity (*h*; Nei, 1987) and nucleotide diversity (*π*; Tajima, 1983) were also calculated to assess levels of genetic variation within populations.

### 2.6 Population bottlenecks

Genetic evidence for a recent reduction in population size was tested for each population by heterozygosity excess, L‐shaped graph, and M‐ratio methods (Cornuet & Luikart, 1996; Garza & Williamson, 2001; Luikart, Allendorf, Cornuet, & Sherwin, 1998). Heterozygosity excess tests were performed with the program BOTTLENECK 1.2 (Piry, Luikart, & Cornuet, 1999) by the Infinite Allele Model (IAM) and Two‐Phased mutation Model (TPM), incorporating 78% of single‐step mutations and 12% of variance among multiple steps, following the recommendation of Peery et al. (2012). Statistical significance of the number of loci with heterozygosity excess as expected in bottlenecked populations (Luikart et al., 1998) was evaluated by a one‐tailed Wilcoxon signed‐rank test from 10^4^ simulation replicates. The L‐shaped method illustrates the frequency of rare alleles in the populations; an L‐shape graph indicates that the population is in mutation–drift equilibrium (Luikart et al., 1998). The BOTTLENECK software was also used to establish if allele frequency distribution was L‐shaped. Finally, test for signatures of a bottleneck by the M‐ratio method (Garza & Williamson, 2001) was applied using the StrataG package in R (Archer, Adams, & Schneiders, 2016). The M‐ratio statistic indicates the number of unoccupied potential allelic states and was shown to be small (<0.68 in a dataset with seven loci) when a severe population decline had occurred (Garza & Williamson, 2001).

### 2.7 Genetic differentiation and structuring

A hierarchical analysis of molecular variance (AMOVA) was performed on microsatellite allele identity and mtDNA to determine how genetic diversity was distributed within and among subspecies and populations. Statistical significance was determined in ARLEQUIN 3.5.1.3 (Excoffier & Lischer, 2010) with 1,000 permutations.

Genetic differentiation among all pairs of populations with sample sizes ≥ 5 was assessed by calculating pairwise *F*_ST_ and Φ_ST_ values for microsatellite and mtDNA data, respectively. *F*_ST_ was computed between pairs of populations following Weir and Cockerham (1984) and Whitlock (2011), and statistical significance was tested by 10^4^ permutations of genotypes among populations under Bonferroni's correction, using GenoDive (Meirmans & Van Tienderen, 2004). Φ_ST_ indices and tests were calculated in ARLEQUIN 3.5.1.3 (Excoffier & Lischer, 2010).

Assignment tests based on multi‐locus microsatellite genotypes were evaluated using two methods because different clustering approaches may lead to different conclusions (Waples & Gaggiotti, 2006). First, we used a Bayesian genotype clustering procedure in STRUCTURE 2.3.3 (Pritchard, Stephens, & Donnelly, 2000). The admixture model was used with and without the LOCPRIOR setting, which considers sample location, and allows structure to be detected when genetic structure is weak or when the number of loci is small (<20; Hubisz, Falush, Stephens, & Pritchard, 2009). Correlated allele frequencies were assumed (Pritchard et al., 2000). For each value (1–13) of the number of independent genetic clusters or K, we ran 10^6^ iterations 20 times (after a burn‐in of 5 × 10^5^ steps). For choosing the optimal number of clusters, three criteria were used; (1) the log likelihood given *K* (*L*(*K*); Pritchard et al., 2000), (2) the second‐order rate of change of mean log likelihood (∆*K*; Evanno, Regnaut, & Goudet, 2005), and (3) the median value of *L*(*K*). The first two were calculated using STRUCTURE HARVESTER online Web server (Earl & VonHoldt, 2012). The third was calculated using CLUMPAK software (Kopelman, Mayzel, Jakobsson, Rosenberg, & Mayrose, 2015) that was also used to find the optimal individual alignments of replicated cluster analyses and to visualize the results.

Population structure was also explored by performing a Discriminant Analysis of Principal Components (DAPC; Jombart, Devillard, & Balloux, 2010) that does not make any assumptions about HWE or linkage disequilibrium. We used K‐means clustering of principal components for *K* = 1 to 20 and Bayesian Information Criteria (BICs) to assess the optimal number of genetic clusters; the K with the lowest BIC value is the optimum (after Carlen & Munshi‐South, 2020). However, BIC values may continue to decrease below the optimum in case of genetic clines and hierarchical structure (Jombart et al., 2010). Therefore, the rate of decrease in BIC values was visually examined to identify values of K, after which BIC values decreased only subtly (Jombart et al., 2010). DAPC was applied using the Adegenet package 2.1.1 in R (Jombart, 2008).

A hierarchical analysis of the genetic structuring using Bayesian and DAPC procedures was performed at different spatial scales to detect a possible pattern among the less isolated populations. This was performed: (a) in all populations, (b) after removing the much‐differentiated Bermuda population, and (c) after removing the three differentiated genetic groups on Bermuda, Ascension/Fernando de Noronha and Europa.

### 2.8 Network and phylogenetic analysis

A statistical parsimony haplotype network was obtained from mtDNA sequences (123 individuals sampled in 13 populations) using the pegas package 0.11 in R (Paradis, 2010). The haplotype network was built using an infinite site model (Hamming distance) (Templeton, Crandall, & Sing, 1992) to display the maternal connections among populations and subspecies of *P. lepturus*.

We used Mega 7.0.26 (Kumar, Stecher, & Tamura, 2016) to align the sequences of 123 *P. lepturus* and six *P. rubricauda*. The model of DNA substitution was determined using jModelTest 2 (Darriba, Tab oada, Doallo, & Posada, 2012). The Tamura and Nei (1993) model (TN93) with invariant sites (I) and discrete Gamma distribution (G) was selected as the best‐fit model of nucleotide substitution. Maximum likelihood (ML) phylogenetic trees were constructed in Mega 7.0.26 (Kumar et al., 2016) using TN93+I+G model and 1,000 bootstrap replicates. Bayesian phylogenetic (BI) trees were reconstructed from the datasets with the BEAST program 1.10.4 (Drummond, Suchard, Xie, & Rambaut, 2012). The Bayesian analyses were performed using a TN93+I+G model, a constant size coalescent tree prior, and a strict clock model of rate as the tree priors, as well as other default parameters. We estimated the time of the most recent common ancestor (MRCA) for nodes of interest observed in the mtDNA tree also with the BEAST program. To calibrate the molecular clock, we used the estimated divergence time between *P. lepturus* and *P. rubricauda* following Kennedy and Spencer (2004). Accordingly, we calibrated the divergence between the two species with a normally distributed prior with a mean of 4 MYr and a standard deviation of 1 MYr.

Three independent Markov Chain Monte Carlo (MCMC) runs of 5 × 10^6^ generations, with a 10% burn‐in were performed. Posterior distributions for parameter estimates and likelihood scores to approximate convergence were visualized and checked with the effective sample sizes (ESS > 200) using the Tracer program 1.7.1 (Rambaut, Drummond, Xie, Baele, & Suchard, 2018). A maximum clade credibility (MCC) tree was estimated by TreeAnnotator 1.10.4 (Drummond et al., 2012) from the combined output of the three MCMC runs using the LogCombiner program after the removal of the initial trees (10%) as burn‐in. The MCC tree was visualized with the program FigTree 1.4.4 (Rambaut, 2012).

### 2.9 Correlations among genetic, morphometric, and geographic distances

Isolation by distance was tested though the correlations among nuclear and mtDNA genetic distances, morphometric distances, and geographic distances using a Multiple Regression of dissimilarity Matrices (MRM; Goslee, 2010). Since the three morphometric measurements were available for only 10 populations (all except Tahiti, Christmas, and Bird islands), genetic and geographic distance matrices were only calculated for these 10 populations. We used the previously estimated *F*_ST_ matrix of genetic distances to calculate a new matrix based on Slatkin (1993) genetic distances *F*_ST_/(1−*F*_ST_). Differentiation in morphometric measurements among 10 populations was estimated using pairwise Mahalanobis D^2^, calculated with the HDMD package in R (McFerrin, 2013). The Great Circle Distance (WGS84 ellipsoid) method was used to calculate geographic distance between each pair of populations using sp package in R (Pebesma & Bivand, 2005). Multiple regression on scaled distance matrices were performed using the ecodist package (Lichstein, 2007) with 10^4^ permutations.

## 3 RESULTS

### 3.1 Phenotypic variation

**FIGURE 2:**
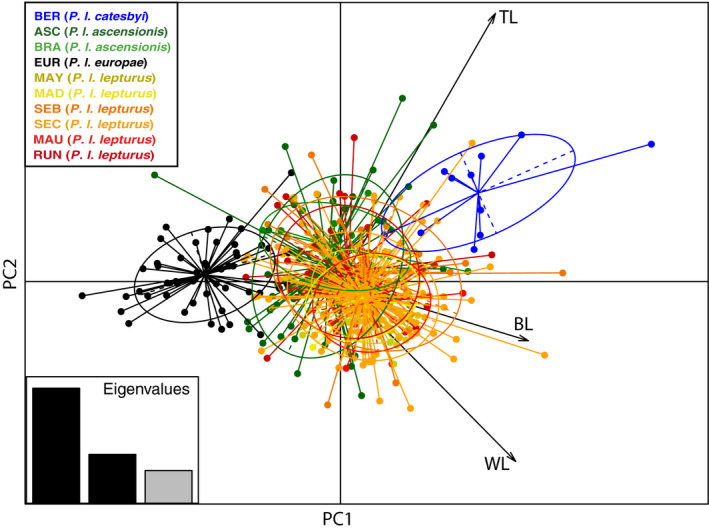
Scatterplot from a principal component analysis (PCA) of morphological variation based on three morphometric measurements [wing length (WL), tarsus length (TL) and bill length (BL)] taken from 421 *Phaethon lepturus* from 10 localities. The total variance explained by PC1 and PC2 was 59% and 25%, respectively. Colors correspond to populations with population codes detailed in Table 2

Based on three morphometric measurements, birds of the subspecies *P. l. catesbyi* (i.e., in the Bermuda population) had significantly longer tarsi than birds in the other populations (Table 3). Birds of the subspecies *P. l. europae* (i.e., in the Europa population) were consistently smaller for all three measured traits compared with the remainder (differences of 25, 6, and 6 mm between Bermuda and Europa populations for wing (all *P*s ≤ .006), tarsus (all *P*s ≤ 4 × 10^–6^), and bill (all *P*s ≤ 9 × 10^–6^) lengths, respectively; Table 3). No birds in the other subspecies differed significantly in size in pairwise comparisons (Table 3). The PCA revealed the separation of individuals along the PC1 axis (explaining 59% of the total variance) into three main clusters corresponding to subspecies *P. l. catesbyi*, *P. l. europae,* and the others (*P. l. ascensionis*, *P. l. lepturus* and *P. l. fulvus*; Figure 2). The populations were significantly different in morphology when tested by parametric MANOVA (Wilk's lambda = 0.33, *df* = 9, *p* < 2.2 × 10^–16^). Pairwise permutation MANOVAs showed that *P. l. catesbyi* and *P. l. europae* were significantly bigger and smaller, respectively, compared to all other populations (all *P*s < 0.05).

**TABLE 3:** Mean morphometric measurements (±1 *SD*) of five subspecies of *Phaethon lepturus* in 11 populations (see Table 1 for further details)

Population	Subspecies	Morphometric measurements									
		Wing length (mm)			Tarsus length (mm)			Bill length (mm)			
		*N*	Mean ± 1 *SD*	Pairwise	*N*	Mean ± 1 *SD*	Pairwise		*N*	Mean ± 1 *SD*	Pairwise
Bermuda	*catesbyi*	11	285.55 ± 5.54	a	11	26.95 ± 1.55	a		11	50.37 ± 2.24	a
Ascension	*ascensionis*	13	270.46 ± 7.78	b	13	23.62 ± 0.87	cd		13	48.45 ± 1.75	abc
Brazil (Fernando de Noronha)	*ascensionis*	60	273.25 ± 6.83	bc	60	23.21 ± 1.53	bcd		60	47.78 ± 2.63	b
Europa	*europae*	140	261.43 ± 6.39	f	50	21.33 ± 1.01	e		140	44.51 ± 1.86	d
Mayotte	*lepturus*	40	276.83 ± 5.28	e	40	22.87 ± 0.71	b		40	49.84 ± 1.70	a
Madagascar	*lepturus*	8	279.38 ± 3.29	ade	8	23.00 ± 1.00	bcd		8	49.06 ± 1.92	abc
Seychelles (Bird)	*lepturus*	32	276.75 ± 9.83	bcde	32	23.50 ± 1.31	c		32	49.23 ± 2.71	ac
Seychelles (Cousin)	*lepturus*	230	279.89 ± 7.05	ad	144	23.03 ± 1.07	bd		230	49.27 ± 1.97	a
Mauritius	*lepturus*	25	280.60 ± 5.84	ad	23	23.07 ± 0.87	bcd		24	49.09 ± 1.86	abc
Reunion	*lepturus*	45	276.24 ± 8.30	ce	41	23.35 ± 1.14	bcd		45	47.96 ± 2.07	bc
Christmas	*fulvus*	12	280.25 ± 4.37	ade	0	NA	–		11	49.66 ± 2.13	abc

Note

Significant differences among populations as detected by Wilcoxon's signed‐rank tests are signified by different lowercase letters following the Benjamini and Hochberg (1995) correction for multiple pairwise comparisons and using an alpha threshold of 0.05.

### 3.2 Genetic diversity and test of bottleneck

No null alleles, large‐allele dropout nor stutter peaks were detected for the 10 microsatellite loci. The percentage of missing data was 0.81%. Linkage disequilibrium among loci was detected for three of the 45 loci pairs (*p* < .05) but no significant linkage disequilibrium was observed among any of the loci after the Benjamini and Yekutieli (2001) correction for multiple tests (adjusted *α* = 0.011), suggesting that all loci were independent. The mean allelic richness, based on minimum sample size of 11 individuals, ranged from 3.5 to 6.8 alleles per locus and was relatively similar among populations, except for the less variable Atlantic Ocean and Mozambique Channel populations (Table 4). All studied populations contained one (Ascension, Seychelles [Aride], Mauritius) to nine (Mayotte) private alleles (Table 4). Observed heterozygosity and unbiased expected heterozygosity ranged from 0.28 to 0.61 and from 0.28 to 0.63, respectively (Table 4). Deviations from HWE were not significant for all populations (all *P*s > 0.05).

**TABLE 4:** Estimates of genetic diversity at 10 microsatellite loci and an 872‐bp fragment of the mtDNA COI in 13 populations of *Phaethon lepturus*

Population	Subspecies	Microsatellite data						Mitochondrial control region			
		AL	AR	AP	*H*_o_	*H*_E_	*F*_IS_	*H*	*h*	*π*	Haplotypes (*N*)
Bermuda	*catesbyi*	4.50	3.53 a	4	0.281	0.283	0.010	3	0.750	0.00123	III (3), IV (3), V (2)
Ascension	*ascensionis*	4.20	3.81 a	1	0.474	0.477	0.007	2	0.143	0.00016	I (13), II (1)
Brazil (Fernando de Noronha)	*ascensionis*	2.60	NA	0	0.475	0.446	−0.076	1	0.000	0.000	I (4)
Europa	*europae*	6.40	4.11 a	3	0.508	0.529	0.034	3	0.385	0.00077	VIII (11), IX (2), X (1)
Mayotte	*lepturus*	10.40	6.24 b	9	0.602	0.647	0.065	3	0.603	0.00062	VII (9), XV (1), XVI (3)
Madagascar	*lepturus*	3.90	NA	1	0.575	0.600	0.048	1	0.000	0.000	VII (4)
Seychelles (Bird)	*lepturus*	9.60	6.46 b	2	0.591	0.604	0.022	3	0.524	0.00066	VII (5), XVI (1), XVII (1)
Seychelles (Cousin)	*lepturus*	11.00	6.29 b	3	0.605	0.628	0.042	4	0.667	0.00242	VII (7), XII (2), XVI (2), XVIII (1)
Seychelles (Aride)	*lepturus*	8.10	6.18 b	1	0.608	0.617	0.015	2	0.333	0.00038	VII (5), XVI (1)
Mauritius	*lepturus*	8.10	6.01 b	1	0.580	0.618	0.081	3	0.714	0.00246	VII (2), XI (2), XII (4)
Reunion	*lepturus*	9.30	5.52 b	3	0.609	0.614	0.016	4	0.539	0.00212	VII (2), XII (9), XIII (1), XIV (1)
Christmas	*fulvus*	6.60	5.67 b	2	0.604	0.621	0.027	2	0.167	0.00019	VI (11), VII (1)
Tahiti	*dorotheae*	6.80	6.80 b	3	0.591	0.600	0.017	3	0.464	0.00225	VII (1), XII (6), XVI (1)

Note

AL, mean number of alleles per locus; AR, mean allelic richness per locus based on minimum sample size of 11 diploid individuals [means followed by the same lower case letter are not significantly different (i.e., *p* > .05) according to the pairwise Wilcoxon's signed‐rank tests with Bonferroni correction]; AP, private allelic richness; *H*_O_, observed heterozygosity over all loci; *H*_E_, unbiased expected heterozygosity; *F*_IS_, fixation index of Weir and Cockerham (1984); *H*, number of haplotypes; *h*, haplotype diversity; *π*, nucleotide diversity; *N*, number of birds with each haplotype.

**FIGURE 3:**
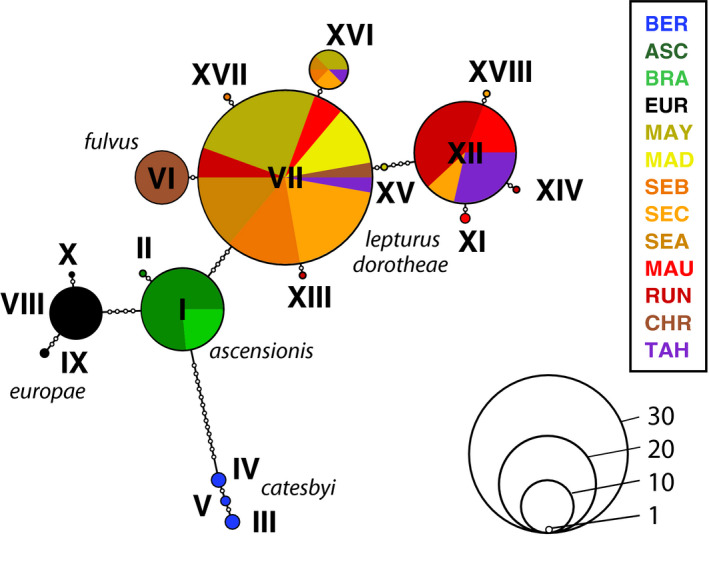
Haplotype network of COI mtDNA sequences (872 bp) from 123 *Phaethon lepturus* sampled in 13 populations. Pie charts and filled circles correspond to haplotypes. Open circles represent mutational steps. Colors correspond to populations with population codes and associated subspecies detailed in Table 2

Eighteen different mtDNA COI haplotypes were obtained from 872 bp COI sequences of 123 individuals sampled across 13 populations (Table 4). Haplotypes were defined by 32 polymorphic sites (3.7%), all of which were substitutions, including 31 transitions and one transversion. Populations of the North (Bermuda) and South Atlantic (Ascension/Fernando de Noronha), and of the Mozambique Channel (Europa) presented three, two and three private haplotypes, respectively (Table 4, Figure 3). One haplotype (VII, 36 individuals) was found in high frequency in all populations of the Indian (except Europa) and Pacific Oceans, but it was represented by a single individual in the Christmas Island population (Table 4). All other individuals from Christmas Island shared a private haplotype (VI). Nucleotide diversity and haplotype diversity were lower in Ascension, Europa, Aride and Christmas populations compared with the other populations (Table 4). Ewens–Watterson (all *P*s > 0.09) and Tajima's D (all *P*s > 0.13) tests within all populations were not significant, providing no evidence of deviations from selective neutrality.

The L‐shaped allele frequency distribution graphs and M‐ratio statistics were not consistent with recent bottleneck events in any population. All populations had normal L‐shaped distributions and M‐ratio statistics well above 0.68. However, heterozygosity excess was detected in Ascension, Europa, Reunion and Mauritius colonies (*P*s < 0.05; Appendix S1, Table S5), suggesting that these populations were not in mutation–drift equilibrium.

### 3.3 Genetic differentiation and population structure

The AMOVA using microsatellite allele identity indicated that 65% of microsatellite variation was attributed to differences among the delineated subspecies, 10% among the populations and 25% among individuals (all *P*s < 0.001). With mtDNA, 73% of the variance was attributed to differences among subspecies, 13% to among populations and 14% to among individuals (all *P*s < 0.001).

#### 3.3.1 Microsatellite differentiation and clustering

Pairwise *F*_ST_ values ranged between 0.003 and 0.485 with a global *F*_ST_ of 0.102 (*p* < .001). Based on *F*_ST_ values, three populations were clearly distinguishable: (1) Bermuda, (2) Ascension, and (3) Europa, suggesting a strong isolation of these three populations (Appendix S1, Table S6). For the Indo‐Pacific populations, *F*_ST_ values indicated weak (maximum value: 0.056), although statistically significant genetic structuring for two‐thirds of the pairwise comparisons and suggested isolation of the Reunion population (all *P*s < 0.05; Appendix S1, Table S6).

**FIGURE 4:**
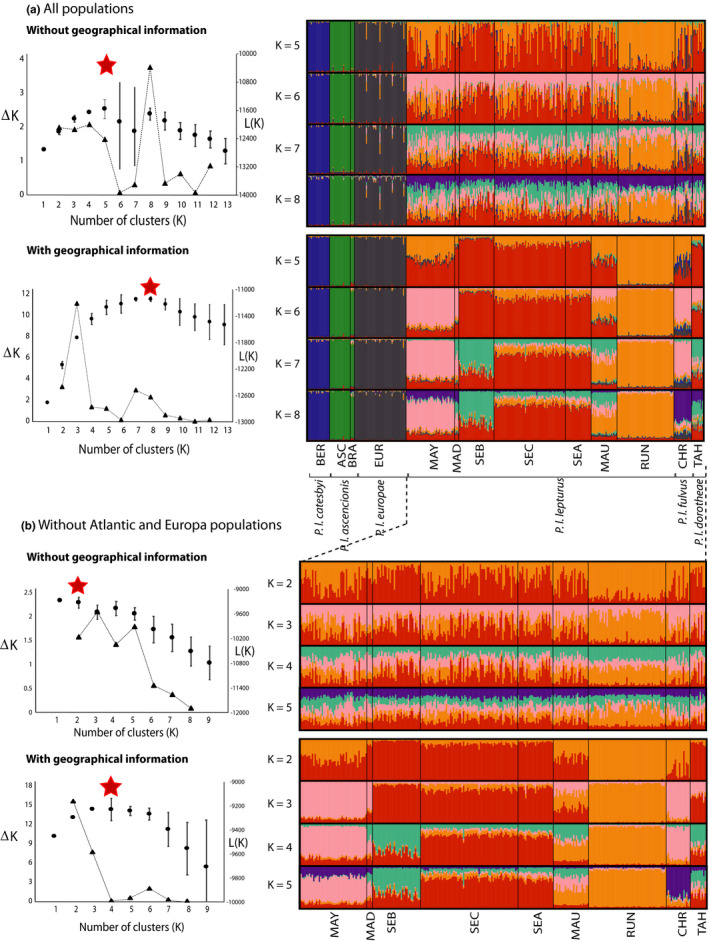
Genetic structure based on 10 microsatellite loci using STRUCTURE with and without geographic information models. Left: Detection of the number of genetic clusters *K* using the log likelihood mean values *L*(*K*) (black circles), median values of *L*(*K*) (red stars) and Δ*K* statistic (after Evanno et al., 2005; black triangles) as derived from STRUCTURE with *K* ranging from 2 to 13 with each value obtained by averaging the posterior probabilities over 20 independent runs. Right: Proportional membership probability of an individual to a given cluster. (a) Analysis of 382 *Phaethon lepturus* from 13 localities and K varied from 5 to 8, and (b) analysis of 287 individuals from nine localities in Indo‐Pacific Oceans (except Europa) and K varied from 2 to 4. Colors correspond to genetic clusters. Population codes and associated subspecies are detailed in Table 2

Clustering of microsatellite genotypes using STRUCTURE analysis showed that the best‐supported model contained three to eight genetic clusters, depending on the optimal K method used (Figure 4a) and suggested a hierarchical structure. Without geographic information, the best‐supported model contained five or eight genetic clusters (break in the slope of Evanno's likelihood values at *K* = 8, maximum *L*(*K*) at *K* = 5 and optimal *K* using median value of *L*(*K*) at *K* = 5; Figure 4a). Clustering analysis using the geographic information model showed that the optimal *K* varied from three (Evanno's procedure) to eight clusters (mean and median *L*(*K*) values; Figure 4a). Regardless of the model and optimal value of *K*, analyses clearly distinguished three clusters corresponding to North and South Atlantic regions (the Bermuda population forming one and the Ascension and Fernando de Noronha populations the second) and the Europa population in the Mozambique Channel (Figure 4a). All the other populations were not clearly isolated. Similar clustering analysis performed after removing (a) the Bermuda population and (b) the Bermuda and Ascension/Fernando de Noronha populations showed the same patterns; distinct clusters in the South Atlantic Ocean and in the Mozambique Channel (data not shown). At the Indo‐Pacific Ocean scale, the optimal number of clusters varied from two to four, depending of the method (Figure 4b). Without geographic information, no clear structure was revealed with all populations appearing admixed. Using geographic information, three pure and genetically distinct clusters (Reunion, Mayotte/Christmas and Seychelles populations) and two admixed populations (Mauritius, Tahiti) could be distinguished. Based on these results, the subspecies *P. l. catesbyi*, *P. l. ascensionis,* and *P. l. europae* seemed to be clearly isolated while the others (*P. l. lepturus*, *P. l. fulvus,* and *P. l. dorotheae*) were admixed.

**FIGURE 5:**
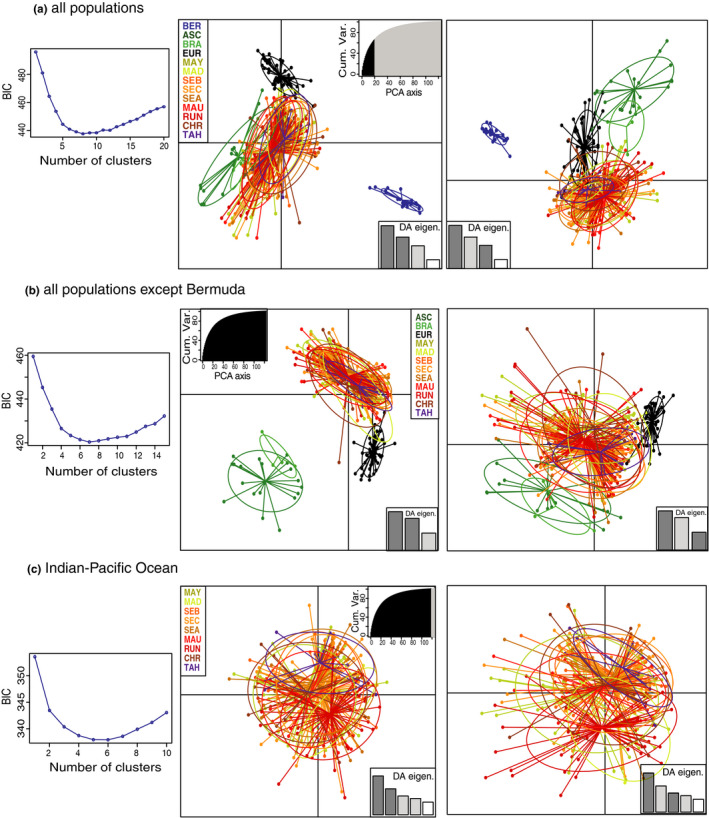
Discriminant analysis of principal components (DAPC) of genetic variation based on 10 microsatellite loci of *Phaethon lepturus*. Individual birds (dots) and populations (ellipses) are discriminated using the first two component axes. Left: Bayesian Information Criterion (BIC) for each K from Adegenet function in R (after Jombart et al., 2010). Right: Scatterplots from DAPC 1st‐2nd axes and 1st‐3rd axes. Percentage of cumulated variance (Cum. Var.) and DA Eigenvalues (DA Eigen.) are plotted. (a) Analysis based on 382 birds from 13 localities. (b) Analysis excluding Bermuda population and based on 361 birds. (c) Analysis at the Indo‐Pacific Ocean scale (except Europa) based on 287 birds. Colors correspond to populations whose codes are detailed in Table 2

The DAPC clustering produced similar estimates of optimal number of clusters as the previous method. The initial sharp decline in BIC values continued up to *K* = 5–8 for all populations, *K* = 4–6 for all populations except Bermuda, and *K* = 2–5 at the Indo‐Pacific Ocean scale except Europa (Figure 5). The first axis of the DAPC reduced space plot clearly distinguished between the Bermuda population and the others (100% assignment‐success rate, Figure 5a). Removing the Bermuda population, the South Atlantic populations (100% assignment‐success rate) and the Europa population (98% assignment‐success rate) were isolated from the others (Figure 5b). All the populations of the Indo‐Pacific Ocean except Europa were admixed, with a maximum of 71% assignment‐success rate between two clusters (Figure 5c).

#### 3.3.2 mtDNA network and phylogeny

The haplotype network based on mtDNA sequences showed the same pattern of genetic structuring as shown by the microsatellite analyses (Figure 3). The Bermuda population (*P. l. catesbyi*) was the most isolated with 13 nucleotide changes distinguishing it from the Ascension population and three private haplotypes. The Europa population was related only to the Ascension/Fernando de Noronha populations (four substitutions between them) and the Ascension/Fernando de Noronha populations were separated from the Indo‐Pacific populations by four substitutions. The Europa and Ascension‐Fernando de Noronha populations had private haplotypes (three and two, respectively). Pairwise Φ_ST_ values among populations confirmed these findings with all comparisons among these three populations and the remainder yielding Φ_ST_ values > 0.81 which were significantly different from 0 (Appendix S1, Table S6). All other individuals located in Indian and Pacific Oceans exhibited extensive shared haplotypes among populations (Figure 3). The most frequent haplotype (VII) was present in all sampled population of the Indo‐Pacific region except Europa. The population of Christmas Island (i.e., *P. l. fulvus*) contained 92% of individuals with a private haplotype (VI). All comparisons among populations in the Indo‐Pacific region except Europa yielded Φ_ST_ values < 0.77 (and <0.66 if the Christmas Island population was excluded); half of them were not significantly different from 0 (Appendix S1, Table S6).

**FIGURE 6:**
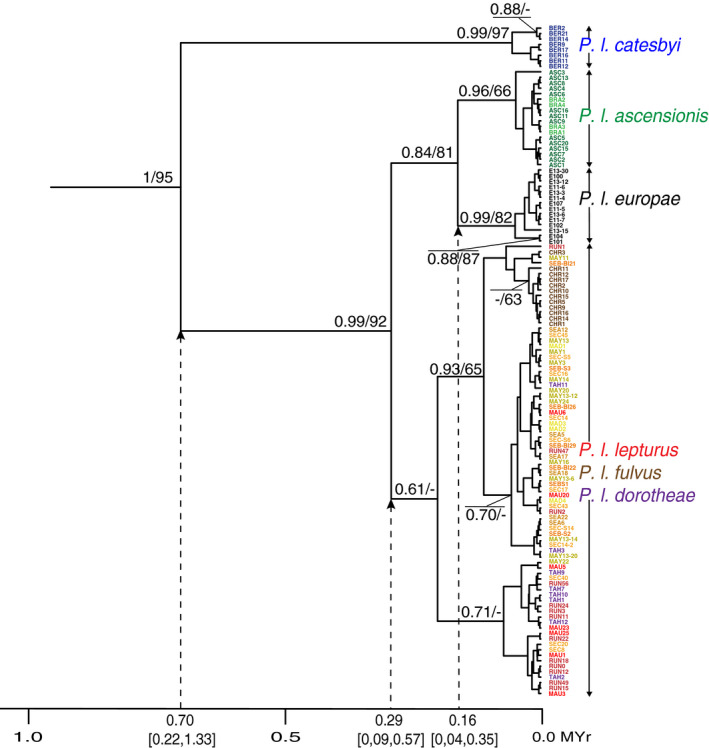
Phylogenetic relationships and estimated divergence times based on COI mtDNA sequences (872 bp) from 123 *Phaethon lepturus* from 13 populations. Statistical support is given above branches; number shown in order for the respective analysis (Beast posterior probabilities/Maximum Likelihood bootstrap values) only if *p* > .50 and bootstrap values >50. Numbers on the x‐axis refer to millions of years (MYr) since the present; mean estimated times in MYr and the 95% intervals are indicated for each major node corresponding to the four phylogroups. Colors correspond to populations whose codes and associated subspecies names are detailed in Table 2

Phylogenetic analysis confirmed the same relationships as in the haplotype network (Figure 6). Both phylogenetic analyses grouped populations of *P. lepturus* in three well‐supported and distinct phylogroups: (1) The first clustered the Bermuda population (*P. l. catesbyi*), (2) the second was divided into two well‐supported phylogroups consisting of the Ascension/Fernando de Noronha (*P. l. ascensionis*) populations and the Europa population (*P. l. europae*), and (3) the third included all of the remaining individuals of Indio‐Pacific Oceans (Figure 6). Each of the phylogroups was further subdivided. Three of them showed strong tree node support. The *P. l. catesbyi*, *P. l. ascensionis*, *P. l. europae* phylogroups were each clustered in two subgroups, with strong to medium tree node support (Figure 6). The other three subspecies were not clearly clustered in distinct clades, except individuals from Christmas Island but showed moderate tree node support and were grouped with three individuals from Reunion, Mayotte, and Seychelles populations.

Divergence time between *P. lepturus* and *P. rubricauda* was estimated by BEAST at 3.70 Mya [CIs: 1.63, 5.76 Mya]. According to our molecular dating, the most recent common ancestor of all white‐tailed tropicbirds lived up to about 700 kya (Figure 6). The *P. l. catesbyi* phylogroup was then isolated from its sister clade from which South Atlantic Ocean, Mozambique Channel and Indian‐Pacific Ocean populations originated. The latter further split approximately 290 kya, separating the phylogroup of South Atlantic Ocean/Mozambique Channel populations, and the phylogroup of the Indo‐Pacific populations. The more recent split separating *P. l. ascensionis* and *P. l. europae* phylogroups was dated at approximately 160 kya (Figure 6).

### 3.4 Correlations among genetic, morphometric, and geographic distances

Genetic distances based on nuclear microsatellites and mtDNA sequences estimated for 10 populations showed significant relationships with geographic distances (MRM tests, *r* = .48, *p* = .001, and *r* = .20, *p* = .012, respectively). Morphometric distances estimated for the same 10 populations were weakly correlated with nuclear genetic distances (MRM test, *r* = .16, *p* = .03) but not with mtDNA genetic distances (MRM test, *r* = .15, *p* = .06).

## 4 DISCUSSION

The present study has provided data on genetic and morphological differences among putative white‐tailed tropicbird subspecies. Patterns of variation in white‐tailed tropicbirds revealed significant differences at different geographic scales and suggested disparities in connectivity among populations. This structuring was broadly in agreement in morphological, nuclear, and mtDNA marker analyses. Our results showed clear morphological and genetic differentiation in some subspecies such as *P. l. catesbyi*, represented by the Bermuda population, in which birds are larger and display a genetic clade potentially isolated from the Middle Pleistocene. Birds of Europa (*P. l. europae*) were the smallest and constituted a highly differentiated genetic cluster. The subspecies *P. l. ascensionis* from the South Atlantic populations is morphologically similar to the Indo‐Pacific populations but genetically isolated. Birds from the remaining populations of the Indo‐Pacific region were morphologically similar and showed weak genetic differentiation. These results are discussed below in terms of subspecies validity and appropriate units for conservation strategies.

### 4.1 Morphological variation among subspecies

Morphometric measurements of birds showed clear differentiation among the larger subspecies *P. l. catesbyi*, the smaller subspecies *P. l. europae* and the other subspecies that were intermediate in size (Table 3 and Figure 2). Our results were not entirely consistent with those of Le Corre and Jouventin (1999) who distinguished between large subspecies (i.e., *P. l. catesbyi*, *P. l. lepturus,* and *P. l. fulvus*) and small subspecies (i.e., *P. l. ascensionis*, *P. l. europae,* and *P. l. dorotheae*). This difference in findings of these two studies may be partly explained by Le Corre and Jouventin (1999) sampling birds of the subspecies *ascencionis* from the São Tomé population as opposed to the present study that included birds from the Ascension and Fernando de Noronha populations. There are slight morphological differences between birds from the western and eastern South Atlantic, with birds from Ascension/Fernando de Noronha populations slightly bigger than birds from the São Tomé population (see table 2 in Le Corre and Jouventin (1999) and Table 3 in the present study).

As previously reported by Le Corre and Jouventin (1999), body size appeared to decrease from the highest (Bermuda, 32°N) to the lowest (Europa, 22°S) latitudes but variation was only subtle with none of the correlations of any morphological traits with latitude or longitude being statistically significant (data not shown). Body size variation of *P. lepturus* could be explained, in part, by Bergmann's Rule, applied at an intraspecific level, as suggested by Le Corre and Jouventin (1999). Phenotypic structuring results from the selection of multiple pressures to which an organism is exposed (Mayr, 1956), such as differences in local oceanographic characteristics around distinct seabird populations (Friesen, 2015). Environmental characteristics (e.g., sea surface temperature, primary productivity) and characteristics relating to foraging behaviour seem to be correlated with the distribution of phenotypes in the marine realm, as has been suggested for seabirds (Weimerskirch, Zimmermann, & Prince, 2001; Yamamoto et al., 2016).

### 4.2 Genetic structuring among subspecies

Microsatellites and mtDNA analyses suggested complex structuring among white‐tailed tropicbirds subspecies. *Phaethon l. catesbyi* represented by the Bermuda population was the most ancient and the most isolated group, estimated to have been potentially isolated since the Middle Pleistocene. The subspecies *P. l. ascensionis* (South Atlantic populations) and *P. l. europae* (South Mozambique Channel) were each isolated and may have a common ancestor. Birds from the remaining subspecies (*P. l. lepturus*, *P. l. fulvus,* and *P. l. dorotheae* represented by only one population) of the Indo‐Pacific region showed no clear and weak genetic differentiation but sample sizes were generally low.

Birds from Bermuda are the most divergent and isolated genetic group, as suggested by microsatellite clustering (Figure 4), DAPC (Figure 5), the mtDNA network (Figure 3), and phylogeny (Figure 6). This subspecies could have diverged from others approximately 700 kya (Figure 6). Several factors could explain this strong isolation. Mejías, Wiersma, Wingate, and Madeiros (2017) showed that the postbreeding dispersion of the Bermudian white‐tailed tropicbirds extended as far west as North Carolina and as far east as the Mid‐Atlantic Ridge. None of the tracked birds crossed the equator and reached the southern Atlantic Ocean. Campos, Andrade, Bertrand, and Efe (2018) and Santos, Campos, and Efe (2019) tracked the movement of white‐tailed tropicbirds (*P. l. ascensionis*) during chick‐rearing from Fernando de Noronha archipelago and showed that long foraging trips reached an average distance from the breeding colony of 105 ± 48 km. This reinforces the hypothesis of a lack of connectivity between northern and southern populations of the Atlantic Ocean. Indeed, birds from Bermuda are genetically and morphologically distinct from birds of Ascension and Fernando de Noronha, suggesting that gene flow does not occur between the southernmost and northernmost populations of the Atlantic Ocean. Bermuda and Ascension Islands are separated by 39° of latitude (or 7,800 km), and so it is likely that latitudinal distance plays a major role in their genetic isolation. There is a pressing need to investigate the genetic structure of birds along the southeast–northwest gradient of the tropical Atlantic (including Great and Lesser Antilles), to identify the spatial limits of northern and southern genetic clusters.

The phylogroup of *P. l. ascensionis* and *P. l. europae* could have a common ancestor that probably diverged from the ancestor of the Indo‐Pacific region about 290 kya (Figure 6). All nuclear microsatellite analysis showed a strong isolation for each of these two populations. The geographic localization of the ancestor could not be determined and would not have been reliable with only one sequence of <1,000 bps carried a tiny amount of phylogenetic signal. Nevertheless, sporadic gene flow could be possible between Atlantic and Indian Oceans. Some birds of the tropical Atlantic (i.e., from Ascension and Fernando de Noronha, but also from São Tomé and Príncipe in the Gulf of Guinea—not included in our study) may have migrated to the Indian Ocean, probably through the southern tip of Africa. Similar gene flow between Atlantic and Indian Oceans was proposed for petrels (*Pterodroma* spp.; Booth Jones et al., 2017; Brown et al., 2010) and between Atlantic and Pacific Oceans for magnificent frigatebirds *Fregata magnificens* (Hailer et al., 2010).

Birds of Europa Island were genetically isolated from all other populations of the western Indian Ocean, indicating very limited gene flow between Europa and the other populations of the region. Ecological barriers to gene flow are unlikely because there is no marked marine ecological gradient between the northern and southern Mozambique Channel nor between the west and east of Madagascar. Movements of white‐tailed tropicbirds from Europa have not been studied to date but we have no indirect evidence of any regional dispersal with no ringing recoveries outside of Europa. However, the nonbreeding distribution of several seabird species breeding in the Mozambique Channel has been studied and all of them are capable of crossing the Mozambique Channel or of flying south of Madagascar to reach the Mascarene archipelago (Le Corre et al., 2012; Weimerskirch et al., 2006). The isolation of Europa Island tropicbirds also manifests itself in at least two other marine vertebrates for which a role for ecological isolation is suspected. Marine conditions in the southern and central Mozambique Channel are thought to drive genetic isolation of local populations of the green turtles *Chelonia mydas* (Bourjea et al., 2007). The red‐footed booby *Sula sula* is a polymorphic pantropical seabird and most populations in the Indian Ocean predominantly consist of the white‐tailed white morph except at Europa Island where >95% are of the white‐tailed brown morph, suggesting strong isolation (Le Corre & Jouventin, 1999) as confirmed by a recent genetic study (Danckwerts, 2018). We suggest that the strong genetic isolation of *P. l. europae* on Europa Island may be due to several factors such as natal philopatry and breeding fidelity, breeding phenology, ecological specialization, and sexual selection (Friesen, Burg, & McCoy, 2007; Lombal et al., 2020; Sexton, Hangartner, & Hoffmann, 2014; Uy, Irwin, & Webster, 2018).

Surprisingly, the only population of the Pacific Ocean that we included in our study (Tahiti, French Polynesia) was genetically indistinguishable from populations of the Indian Ocean (except birds in the Europa population). The lack of nuclear and mtDNA genetic differentiation between Indo‐Pacific subspecies (i.e., *P. l. lepturus* and *P. l. dorotheae*, represented by one breeding population in Tahiti) demonstrates that the Indonesian archipelago is not a physical barrier to seabird dispersion, suggesting that gene flow occurs among populations in the two ocean systems. An alternative scenario that would explain this result would be from only recent isolation of a previously panmictic population. An ongoing regional study of migration strategies of 10 seabird species of the western Indian Ocean has shown that some species, such as Lesser frigatebirds (*Fregata ariel*), sooty terns (*Onychoprion fuscatus*), and red‐tailed tropicbirds (*Phaethon rubricauda*), occasionally fly over the Timor Sea and the Indonesian straits to reach the Pacific Ocean (Jaeger et al., 2017; Le Corre et al., 2012; Weimerskirch et al., 2017; MLC unpublished data). Preliminary results from migration studies of white‐tailed tropicbirds in the western Indian Ocean (from Mayotte and Cousin Island, Seychelles) suggest that birds undertake large‐scale eastward migrations toward the central and eastern Indian Ocean (Le Corre et al., 2012). Birds of the three tracked populations reached the longitude 90°E and one bird from Mauritius even reached the western coast of Sumatra (MLC unpublished data). It is possible that white‐tailed tropicbirds disperse widely over many degrees of longitude. To explore this further, population structuring and genetic connectivity along the north–south gradient of the Pacific Ocean, including populations between Australia and Tahiti, would be of great interest. Including populations covering the entire range of *P. l. dorotheae* would help to verify the morphological and genetic similarities between *P. l. lepturus* and *P. l. dorotheae*. Our data only allowed us to consider the genetic relationships between the Tahiti population and *P. l. lepturus*.

### 4.3 Implications for subspecies validity and conservation

Our findings question the validity of classifying white‐tailed tropicbirds into six distinct subspecies and help to define appropriate conservation units (Frankham, 2010; Haig et al., 2011; Taylor & Friesen, 2012). The results of AMOVA tests using microsatellite and mtDNA markers confirmed that subspecies designation explained the global genetic variation of the species at large better than did the population designation. Variation among the delineated subspecies was high (i.e., >65% for both markers), but only a low percentage (i.e., 10%–13%) of the variation could be attributed to differences among populations.

#### 4.3.1 Isolated and threatened subspecies: *P. l. catesbyi*, *P. l. ascensionis,* and *P. l. europae*

Both nuclear and mtDNA data identified three distinct lineages of birds on Bermuda, Ascension/Fernando de Noronha and Europa, corresponding to *P. l. catesbyi*, *P. l. ascensionis,* and *P. l. europae* subspecies, respectively. Each lineage was restricted to its own island with no co‐occurrence of lineages on the same island. These three subspecies should be maintained and be defined as a priority for conservation. Low genetic diversity indices (Table 4) and probably recent population size reduction showed evidence of historic genetic bottlenecks (Friesen, Gonzalez, & Cruz‐Delgado, 2006; Lombal et al., 2020; Morris‐Pocock, Hennicke, & Friesen, 2012), particularly in *P. l. europae* and *P. l. ascensionis* at Ascension (Table 4) and at Fernando de Noronha (see Nunes et al., 2017).

The Bermuda genetic group probably includes all populations of the northwest tropical Atlantic Ocean (i.e., *P. l. catesbyi*). Lee and Walsh‐McGehee (2000) reported that the subspecies had declined in regional population size by 50% since 1984, probably due to loss of breeding habitats and predation by invasive mammals. They estimated that the region contained 5,500 pairs, of which 2,500 (or 45%) breed in Bermuda, 1,000 (or 19%) in the Bahamas, 1,000 in Hispaniola and the Greater Antilles and with the remainder (or 17%) breeding in 20 small populations. Because the species is highly philopatric, individuals are unlikely to relocate their breeding sites to avoid further threats (Wingate & Talbot, 2003). Their breeding success (as measured by the percentage of nests regularly visited by adults that fledge a chick) decreased from 66.6% in 1970–1983 to 48.8% in 2001–2002 (Wingate & Talbot, 2003). The nonsignificant tests of genetic bottleneck could be unreliable due to small sample sizes (Piry et al., 1999). *Phaethon lepturus catesbyi* should be regarded as vulnerable because of low allelic richness (Table 4), the small number and size of populations, and the subspecies decline (Lee & Walsh‐McGehee, 2000). Various conservation interventions should include control or eradication of invasive predators (especially rats and domestic cats), and restoration of breeding habitats.

*Phaethon lepturus ascensionis* includes all populations of the southern Atlantic Ocean (i.e., Abrolhos, Fernando de Noronha, Ascension Island, São Tomé, and Príncipe). This subspecies is particularly at risk on the Brazilian islands where populations are small, threatened by introduced predators and exhibiting low genetic diversity and distinctiveness between the tiny yet discrete populations at Abrolhos and Fernando de Noronha (Nunes et al., 2017). Nunes et al. (2017) estimated the effective population size of Fernando de Noronha to be approximately 100 birds. A recent genetic bottleneck was detected in the Ascension population using the heterozygosity excess method (Appendix S1, Table S5) but not using the M‐ratio. Such method‐related differences have important implications for assessing the timing of population declines with such bottleneck tests (Peery et al., 2012). In principle, the population decline occurred more recently based upon heterozygosity excess estimates. The total population size of this subspecies may be <2,000 pairs (Abrolhos—10 pairs; Fernando de Noronha—200 pairs; Nunes et al., 2017), Ascension Island (1,100 pairs; Ratcliffe et al., 2010), and Príncipe (50–100 pairs) and São Tomé (unknown number of pairs; Bollen, Matilde, & Barros, 2018). These populations are threatened by introduced predators (cats, rats, and tegus *Salvatore merianae*; Nunes et al., 2017). In Brazil, white‐tailed tropicbirds are listed as “Threatened” on the Brazilian Red List (Efe et al., 2018). Because of small population sizes and the low number of breeding colonies in the subspecies *P. l. ascensionis*, we consider this conservation unit to be threatened.

Due to the distinct genetic nature of *P. l. europae*, the Europa Island population should be regarded as a single‐island endemic unit. This subspecies was described by Le Corre and Jouventin (1999) on the basis of its morphometric measurements and plumage color. Microsatellites and mtDNA analyses confirm that *P. l. europae* is genetically isolated from all other populations, and the mtDNA phylogenetic tree also supports the hypothesis that this population may have been founded from a common ancestor shared with the *P. l. ascensionis* group (Figure 6). Genetic diversity of *P. l. europae* was low (Table 4) and reduction in population size was detected using the heterozygosity excess tests under the IAM model (Appendix S1, Table S5). Europa was estimated to contain >1,000 pairs of birds in 1974 (Barré & Servan, 1988), 500–1,000 pairs in 1997 (Le Corre & Jouventin, 1997), and probably < 500 pairs in 2017 (Amy, TAAF, MLC personal communication). Therefore, the population is declining rapidly and conservation measures are needed urgently to reverse this trend. The main threat is predation of eggs and chicks by introduced black rats that are abundant on Europa Island (Russell, Ringler, Trombini, & Le Corre, 2011). Since 1995, breeding success of both red‐tailed and white‐tailed tropicbirds on Europa is consistently <10% and in some years the latter experience entire breeding failure (Le Corre & Jouventin, 1997; Ringler, Russell, & Le Corre, 2015). With such low productivity, annual recruitment of new adults is likely to be close to zero and this undoubtedly will result in local population extinction in the near future. Restriction to a single‐island, rapid population decline and present‐day threats from introduced mammals place *P. l. europae* in the critically endangered category and stress the urgent need for conservation measures.

#### 4.3.2 Species complex of *P. l. lepturus*, *P. l. dorotheae,* and *P. l. fulvus*

The separation of *P. l. lepturus* from *P. l. dorotheae* and *P. l. fulvus* is more questionable than the Atlantic and Mozambique Channel subspecies. Population genetic structure analysis showed that *P. l. fulvus* was inseparable from *P. l. lepturus* although this population contains an unusual color morph that might be the result of phenotypic plasticity (Price, 2006). Moreover, when we consider mtDNA, *P. l. fulvus* possesses a private haplotype (Table 4 and Figure 3), suggesting an original maternal lineage. However, the phylogenetic tree did not clearly isolate the population as unique phylogroup (Figure 6). Our results do not support the validity of *P. l. fulvus* subspecies. The bottleneck test showed no variation in population size (Appendix S1, Table S5) but analysis of a larger sample size is therefore strongly recommended. The population contains an estimated 6,000–12,000 pairs (BirdLife International, 2018) and is not threatened, although introduced predators and historic deforestation may have impacted the species as it has on all endemic avifauna on Christmas Island (James & McAllan, 2014). Even with the validity of *P. l. fulvus* subspecies not support by our findings, the Christmas Island population with a differentiated genetic pool may at the very least be considered as a distinct conservation unit and it should merit further study.

Data from morphometric comparisons, population genetic structuring, and phylogenetic analyses indicate that there is no distinction between *P. l. lepturus* and *P. l. dorotheae*, represented by the Tahiti population, suggesting that the latter may not be a valid subspecies or the Tahiti population may be considered as the same subspecies as birds in east Indian Ocean populations. Although some of these populations may be threatened (especially by invasive predators), the subspecies *P. l. lepturus* appears not to be at risk currently. They include hundreds of populations, and tens of thousands of breeding pairs. Furthermore, different populations seemed to be genetically connected, implying a functional metapopulation of birds (Figures 5 and 6). This means that local population declines may be compensated for by immigrations of birds from other populations. Dispersion in a metapopulation helps to maintain overall viability by rescuing local populations from potential extinction (Akçakaya, Mills, & Doncaster, 2007), also providing hybrid vigor and preventing inbreeding depression (Saccheri & Brakefield, 2002). The mean allelic richness per locus of the studied populations of *P. l. lepturus* subspecies and the Tahiti population ranged from 5.5 to 6.8, which was higher than the allelic richness of all other conservation units of the species (Table 4) and indicates a potential for adaptive evolution of these colonies. None of these populations appeared to have experienced genetic bottlenecks, except for the Mauritius population (Appendix S1, Table S5), for which a recent founder effect is proposed.

Distinction between *P. l. lepturus*, *P. l. dorotheae,* and *P. l. fulvus* subspecies remains unclear from our genetic analyses. Our findings do not allow us to propose taxonomic revision currently but further research, especially in the Pacific Ocean, would allow us to determine if the *P. l. dorotheae* subspecies contains all Pacific populations or if some divergence and isolation mechanisms have occurred.

Defining appropriate conservation units is the most important contribution of population genetics research to conservation. Our study suggests five conservation units for white‐tailed tropicbirds based on our genetic data: (1) Bermuda (and all populations of the northwest Atlantic Ocean); (2) Ascension/Fernando de Noronha (and all populations of the southern tropical Atlantic Ocean); (3) Europa; (4) Christmas Island; and (5) the other Indo‐Pacific colonies. The first three conservation units are clearly vulnerable (*P. l. catesbyi*), endangered (*P. l. ascensionis*) or critically endangered (*P. l. europae*), especially under the enduring threats from invasive animal species. They warrant targeted and urgent scientific attention leading to conservation actions.

## CONFLICT OF INTERESTS

None declared.

## AUTHOR CONTRIBUTION

**Laurence Humeau:** Conceptualization (equal); Formal analysis (equal); Funding acquisition (equal); Investigation (equal); Methodology (equal); Project administration (equal); Software (equal); Supervision (equal); Validation (equal); Writing‐original draft (equal); Writing‐review & editing (equal). **Matthieu Le Corre:** Conceptualization (equal); Funding acquisition (equal); Project administration (equal); Resources (equal); Supervision (equal); Writing‐original draft (equal); Writing‐review & editing (equal). **S. James Reynolds:** Methodology (equal); Resources (equal); Validation (equal); Writing‐original draft (equal); Writing‐review & editing (equal). **Colin Wearn:** Resources (equal). **Janos C. Hennicke:** Funding acquisition (supporting); Resources (equal). **James C. Russell:** Resources (equal); Writing‐original draft. **Yann Gomard:** Investigation (equal). **Hélène Magalon:** Formal analysis; Writing‐original draft; Writing‐review & editing. **Patrick Pinet:** Resources (equal). **Pauline Gélin:** Investigation. **François‐Xavier Couzi:** Resources (equal). **Etienne Bemanaja:** Resources (equal). **Vikash Tatayah:** Resources (equal). **Bacar Ousseni:** Resources (equal). **Gérard Rocamora:** Resources (equal); Writing‐review & editing. **Patrick Talbot:** Resources (equal). **Nirmal Shah:** Resources (equal). **Leandro Bugoni:** Funding acquisition (supporting); Resources (equal); Writing‐review & editing (equal). **Denis Da Silva:** Investigation. **Audrey Jaeger:** Formal analysis (equal); Resources (equal); Validation (equal); Writing‐original draft (equal); Writing‐review & editing (equal).

## Supporting information

Appendix S1

## Data Availability

Morphometric measurements, microsatellite data and mtDNA sequences with GenBank accession numbers: KY784127–KY784144 for *P. lepturus* and MT073267–MT073268 for *P. rubricauda* files are available in the Appendix S1 (Tables S2–S4; DRYAD https://doi.org/10.5061/dryad.9w0vt4bcm).
